# Nb_2_O_5_/g-C_3_N_4_ Composite Photocatalysts Supported on Etna-Derived Aluminosilicate for Solar H_2_ Production

**DOI:** 10.3390/ma19112240

**Published:** 2026-05-26

**Authors:** Roberto Fiorenza, Roberta Chiarenza, Sebastiano Arcidiacono, Eleonora La Greca, Anna Lucia Pellegrino, Maria Teresa Armeli Iapichino, Giuliana Impellizzeri, Marisa Giuffrida, Marco Viccaro, Cristina Maria Belfiore, Salvatore Scirè, Leonarda Francesca Liotta

**Affiliations:** 1 Department of Chemical Sciences, University of Catania, V.le A. Doria 6, 95125 Catania, Italy; robi.chiarenza99@gmail.com (R.C.); se_by@hotmail.it (S.A.); eleonoralagreca@cnr.it (E.L.G.); annalucia.pellegrino@unict.it (A.L.P.); maria.armeliiapichino@phd.unict.it (M.T.A.I.); sscire@unict.it (S.S.); 2 C.I.R.C.C. (Interuniversity Consortium in Chemical Reactivity and Catalysis), UdR of Catania, V.le A. Doria 6, 95125 Catania, Italy; 3 Institute for Microelectronics and Microsystems (IMM), National Research Council (CNR), Via S. Sofia 64, 95123 Catania, Italy; giuliana.impellizzeri@ct.infn.it; 4 Institute for the Study of Nanostructured Materials (ISMN)-CNR, Via Ugo La Malfa 153, 90146 Palermo, Italy; 5 INSTM UdR of Catania, V.le A. Doria 6, 95125 Catania, Italy; 6 Department of Biological, Geological and Environmental Sciences, University of Catania, Corso Italia, 57, 95129 Catania, Italy; marisa.giuffrida@unict.it (M.G.); marco.viccaro@unict.it (M.V.); cristina.belfiore@unict.it (C.M.B.); 7 National Institute of Geophysics and Volcanology (INGV)-Section of Catania, Etnean Observatory (INGV-OE), Piazza Roma 2, 95125 Catania, Italy

**Keywords:** volcano ash, Etna, H_2_ production, niobium oxide, carbon nitride, solar photocatalysis

## Abstract

In this work, Etna ash-derived photocatalysts were investigated for the first time for solar H_2_ production. Volcanic ash, commonly treated as a special waste in eastern Sicily (Italy), was modified through chemical treatment followed by microwave-assisted crystallization, avoiding the conventional high-temperature thermal route. The obtained material was tested both as a bare photocatalyst and as a support for a Nb_2_O_5_/graphitic carbon nitride composite prepared by a hydrothermal method. The Etna-derived photocatalyst exhibited a solar H_2_ production rate (by TEOA photoreforming) of 920 μmol/g_cat_∙h. Upon incorporation of the Nb-based composite, the H_2_ evolution rate increased by about 2.5 times, reaching 2370.5 μmol/gcat∙h, demonstrating a strong synergistic effect. Notably, the developed materials largely outperformed commercial TiO_2_ P25 (25 μmol/g_cat_∙h). The enhanced photocatalytic activity was attributed to the tailored modifications of Etna ash, which increased porosity and promoted aluminosilicate framework reorganization, favoring an optimal distribution of the photocatalytically active TiO_2_ and iron oxide phases. The obtained Nb oxide/carbon nitride supported on modified Etna ash also showed a remarkable stability after six consecutive runs of solar photocatalytic H_2_ production. This work demonstrates a sustainable strategy for converting volcanic waste into efficient multifunctional photocatalysts while minimizing the use of critical raw materials.

## 1. Introduction

The recent COVID-19 crisis and the Russian–Ukrainian war, along with the continuous depletion of the fossil fuel-based energy/economy, have raised serious concerns about the availability of energy and material resources.

The European Union (EU), within the framework of the Critical Raw Materials Act, has defined a set of critical and strategic raw materials necessary to support sustainable development [1]. In this context, the valorization of waste with the development or use of bio-based derived materials can be a valuable solution to reach overall sustainability [2]. Recently, the use of clay materials has gained considerable importance due to their textural and mechanical properties, natural abundance, low cost, minimal energy requirements for processing and high versatility in various fields, such as catalysis and related environmental applications [3].

In this context, volcanic ash in regions with active volcanoes is classified as a critical and special waste. Moreover, it can cause significant problems for both the population and local authorities, which are responsible for its removal and storage.

In eastern Sicily, the abundant availability of volcanic ash, resulting from the continuous eruptive activity of Mt. Etna, has prompted its exploration as a resource rather than a waste. For instance, it has been investigated as a plastic component in the manufacture of ceramic tiles [4] and tentatively transformed into porous ceramics by the cold sintering process [5,6].

The peculiar composition of the Etna ash, together with the presence of several inorganic oxides such as SiO_2_, Al_2_O_3_, TiO_2_, CaO, iron oxides, etc. [7], pointed to the possibility of treating it as a sustainable raw material for the synthesis of aluminosilicate-based mixed oxides catalysts, with the advantage of drastically reducing the cost of the raw materials, synthetizing at the same time a green catalyst with high-added value.

Meanwhile, the demand for green energy and the efficient exploitation of renewable sources has become an urgent priority [8]. Solar photocatalytic H_2_ production represents a sustainable approach for generating this important energy vector via a photocatalytic mechanism. In this process, a semiconductor oxide irradiated with solar light generates electron–hole pairs, with electrons (e^−^) promoted to the conduction band (CB) and holes (h^+^) left in the valence band (VB). The photogenerated holes can oxidize water and/or an organic sacrificial agent (such as triethanolamine, TEOA)—typically added to enhance e^−^/h^+^ separation—producing protons that are subsequently reduced by the electrons in the CB to generate H_2_ [9,10,11].

Based on these considerations, this work reports for the first time the photocatalytic performance of Etna-derived aluminosilicate-mixed oxide materials obtained through chemical treatment coupled with microwave-assisted crystallization of volcanic ash. The developed materials were investigated both as bare photocatalysts and as active supports for Nb_2_O_5_/g-C_3_N_4_ composites in solar H_2_ production. In recent years, niobium oxide (Nb_2_O_5_), which exhibits photocatalytic properties comparable to those of TiO_2_-based systems [12], has been widely combined with sustainable materials such as graphitic carbon nitride (g-C_3_N_4_) [13,14]. This approach allows a reduction in the amount of Nb employed in photocatalytic composites, considering that Nb, similarly to Ti, is included in the EU list of critical raw materials [15]. Moreover, g-C_3_N_4_ is a low-cost and non-critical material easily synthesized from sustainable precursors such as urea or melamine, and its two-dimensional structure favors efficient charge carrier separation, enhancing photocatalytic H_2_ evolution [16].

In this context, the originality of the present work lies in the valorization of volcanic waste as a raw material for photocatalyst synthesis through an energy-saving microwave-assisted crystallization route, replacing conventional thermal treatments. The obtained materials were structurally, texturally, and optically characterized and subsequently evaluated in solar H_2_ production via TEOA photoreforming. To the best of our knowledge, this is the first study to investigate Etna-derived materials both as standalone photocatalysts and as a support for a Nb_2_O_5_/g-C_3_N_4_ composite, highlighting the overall sustainability of the proposed approach for green hydrogen production.

## 2. Materials and Methods

### 2.1. Sample Preparation

All the chemical reagents were used as purchased without further purification.

#### 2.1.1. Etna Ash Treatments

The volcanic ash powders used in this work derived from the eruption of 24 February 2021 at the South East Crater of Mt. Etna, which was one of the most energetic eruptions to have occurred at the volcano in recent times [17]. The chemical analysis furnished by the Department of Biological, Geological and Environmental Sciences of the University of Catania is reported in Table S1.

In order to obtain an aluminosilicate-based mixed metal oxide structure, the ash was treated by employing the procedure used for synthetic fly ash to form Na-based aluminosilicates [18]. In detail, 7.5 mL of 20% *w*/*w* HCl was mixed with 500 mg of Etna ash in order to obtain an acid/ash ratio of 15 mL/g of ash [18]. At this ratio, as reported, the acid treatment favors the formation of aluminosilicate-like structures with a higher Si and Al content than the raw ash, reducing at the same time the concentration of iron and alkaline oxides [19]. The mixture was stirred at 80 °C for 2 h. Afterwards, the solid was filtered and washed until a neutral pH was achieved, then dried overnight at 90 °C. The obtained powders were mixed and grinded with anhydrous sodium hydroxide pellets in a NaOH/ash ratio of 1.25. This procedure promoted the formation of Na-type aluminosilicates [18]. Later, the mixture was fused at 550 °C for 1 h in a muffle, according to the reported procedures [18,20]. Then, the crystallization was promoted by a microwave treatment using an Anton Paar Monowave 200 instrument instead of the conventional thermal process in static conditions. The powders were treated in water at 70 °C (microwave heating ramp of 5 °C/min) for 3 h. Finally, the obtained sample was filtered, washed and dried overnight at 105 °C. This sample was coded as EtnaMW.

#### 2.1.2. Nb_2_O_5_-gC_3_N_4_ Synthesis

The Nb_2_O_5_-gC_3_N_4_ composites were prepared by hydrothermal method.

In detail, the bare Nb_2_O_5_ was synthetized following the procedure reported in ref. [21]. A quantity of 0.5 g of NbCl_5_ was mixed with 20 mL of deionized water and 0.5 mL of concentrated nitric acid (HNO_3_)_._ Then, 10 mL of H_2_O_2_ (3% *v*/*v*) was added. The white suspension became yellow due to the presence of the water-soluble niobium peroxo-complex [Nb(O_2_)_4_]^3-^ species [21]. The resultant solution was then hydrothermally treated in a Teflon-sealed autoclave at 120 °C for 24 h, and the resulting white powder was filtered under vacuum, washed several times and then dried at 100 °C. Finally, the sample was calcined at 200 °C for 2 h (heating ramp: 10 °C/min).

The bare g-C_3_N_4_ (CN) was prepared by thermal polymerization of 5 g of melamine placed in a covered alumina crucible and heated in a muffle at 550 °C for 4 h (heating ramp: 10 °C/min) [22].

Nb_2_O_5_-gC_3_N_4_ composites with weight percentages of Nb_2_O_5_ ranging from 0.5% to 3% wt% were obtained hydrothermally by mixing the as-prepared powders. After mixing, the powders were sonicated in water for 30 min and then transferred to a Teflon-sealed autoclave and treated at 120 °C for 24 h. Finally, the resulting samples were dried overnight at 90 °C.

The samples were coded as NbxCN, where x is the wt% of the graphitic carbon nitride.

#### 2.1.3. Nb_2_O_5_-gC_3_N_4_/Etna-Derived Aluminosilicate Structure Composite

The Nb_2_O_5_-gC_3_N_4_/EtnaMW composite was prepared with a modified solid-state dispersion (SSD) method [23]. The sample labelled Nb2CN being the most active in a preliminary photocatalytic screening (see Section 3.2) was selected. In a typical synthesis, the powders were first mixed using an agate mortar and pestle. Water was then added, and the resulting suspension was sonicated for 30 min and subsequently stirred for 2 h at 70 °C. Finally, the obtained material was dried overnight at 110 °C. This sample was coded as Nb2CN/EtnaMW.

### 2.2. Sample Characterizations

The powder X-ray diffraction (XRD) patterns were registered over a 2θ range of 10–80° using a Rigaku MiniFlex600 powder diffractometer (Rigaku Europe SE, Neu-Isenburg, Germany) equipped with Cu Kα radiation (40 kV, 15 mA). A Ni filter was employed to reduce X-ray fluorescence, and data were collected with a step size of 0.03° and a scanning rate of 1°·min^−^^1^. The crystalline phases were examined according to ICSD files (Inorganic Crystal Structure Database, FIZ Karlsruhe) (Bruker AXS GmbH, Karlsruhe, Germany).

The textural properties of the samples were determined by N_2_ adsorption–desorption at −196 °C using a Micromeritics Tristar II Plus 3020 instrument (Micromeritics Instrument Corp., Norcross, GA, USA) after outgassing the materials overnight at 120 °C. The specific surface area was calculated using the BET (Brunauer–Emmett–Teller) method, while the pore volume and pore size distribution were derived from the desorption branch using the BJH (Barrett–Joyner–Halenda) method.

The UV–Vis DRS (Diffuse Reflectance Spectroscopy) spectra were acquired with a JASCO V-670 (JASCO Europe Srl, Cremella, Italy). It used an integration sphere and BaSO_4_ as the reference material. The optical bandgap of the samples (E_g_) was evaluated by plotting the modified Kubelka–Munk function vs. the hν [24]. Notably, the application of this method to composite materials requires some additional considerations. In accordance with the literature [24,25], indeed, the reflectance spectra of these samples are more complex compared to bare semiconductor oxides with homogenous crystalline phases, considering the absorption from the impurity phases present in this type of complex material. Therefore, to extrapolate the E_g_ values, these features were also considered.

Fourier Transform Infrared (FTIR) spectra were acquired in the range of 4000–400 cm^−1^ using a Perkin Elmer Spectrum Two FT-IR Spectrometer (Perkin-Elmer, Waltham, MA, USA). The background spectrum was obtained using KBr.

The morphological characterization was carried out using a field emission scanning electron microscope (FE-SEM), the ZEISS SUPRA 55 VP (ZEISS, Oberkochen, Germany). Some samples were Au-coated before the FE-SEM characterization in order to ensure conductive behavior. The atomic composition was performed through energy-dispersive X-Ray (EDX) analysis and recorded using an INCA-Oxford windowless detector (Oxford Instruments, Abingdon, UK), with a resolution of 127 eV as the full width half maximum (FWHM) of the Mn Kα.

### 2.3. Solar Photocatalytic H_2_ Production Set-Up

The photocatalytic hydrogen production was investigated under simulated solar irradiation using a solar simulator equipped with an optical fiber (UV large-core optical fiber, transmission: 190–2500 nm (Quantum Design GmbH, Darmstadt, Germany); Xe lamp: 150 W, irradiance of 10.0 mW/cm^2^∙nm) irradiating on the top and inside a home-made Pyrex jacketed reactor maintained at 25 °C. In a typical experiment, 40 mg of powder photocatalyst was added to 40 mL of a water–triethanolamine (TEOA) solution (32 mL and 8 mL, respectively) under constant stirring.

The reaction mixture was purged for 1 h with argon flow to remove the oxygen, and then the solar simulator was turned on for 5 h. The hydrogen production was measured with a gas chromatograph (Agilent 6890; Agilent Technologies, Santa Clara, CA, USA) equipped with a packed column (Carboxen 1000; Supelco Inc., 595 North Harrison Road Bellefonte, PA, USA) and a TCD detector. For the hydrogen quantification, after the opportune standard calibration, a 1 mL aliquot of the reaction gases was withdrawn using a syringe (Hamilton Gastight 1001; Hamilton, Bonaduz AG, Switzerland) and injected in the GC. The photocatalytic tests were carried out in triplicate, and the reported error bars represent the standard deviations of three independent measurements (typical relative error ≈ 3%).

## 3. Results and Discussion

### 3.1. Structural, Morphological, Textural, and Optical Properties

**Figure 1:**
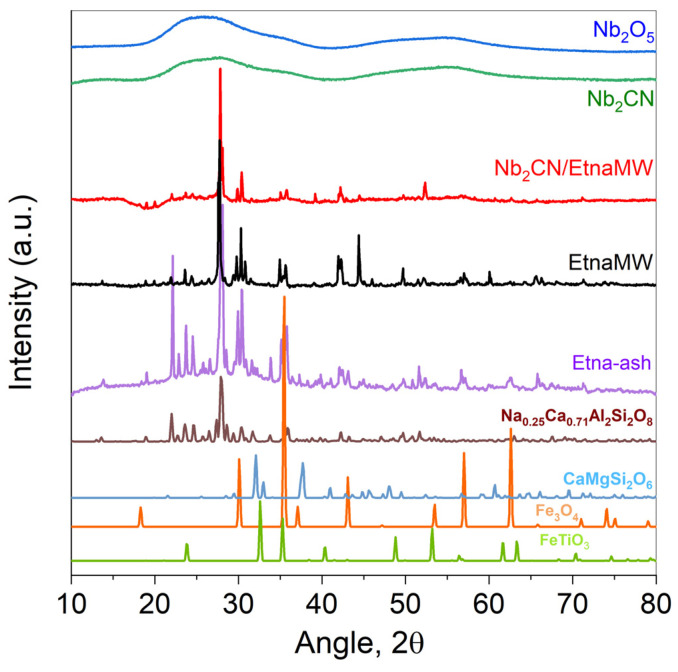
XRD patterns of the investigated samples and reference compounds (Na_0.25_Ca_0.71_Al_2_Si_2_O_8_, CaMgSi_2_O_6_, Fe_3_O_4_ and FeTiO_3_).

Figure 1 reports the XRD patterns of the investigated materials. The Etna ash sample exhibits a complex crystalline profile, typical of a natural volcanic matrix, with the main reflections attributable to a Na-Ca aluminosilicate phase of feldspathic type (plagioclase), in agreement with the reference pattern of Na_0.25_Ca_0.71_Al_2_Si_2_O_8_ (ICSD code #63547). In addition to this main contribution, the presence of further reflections suggests the coexistence of less abundant mineral phases containing Ca, Mg and Fe, ascribable to pyroxene-type silicates (augite—CaMgSi_2_O_6_, ICSD #5205) and, to a lesser extent, Fe and Fe-Ti oxides (Fe_3_O_4_ and FeTiO_3,_ ICSD #26410 and #9805 respectively), which are commonly found in Etna volcanic ashes.

**Figure 2:**
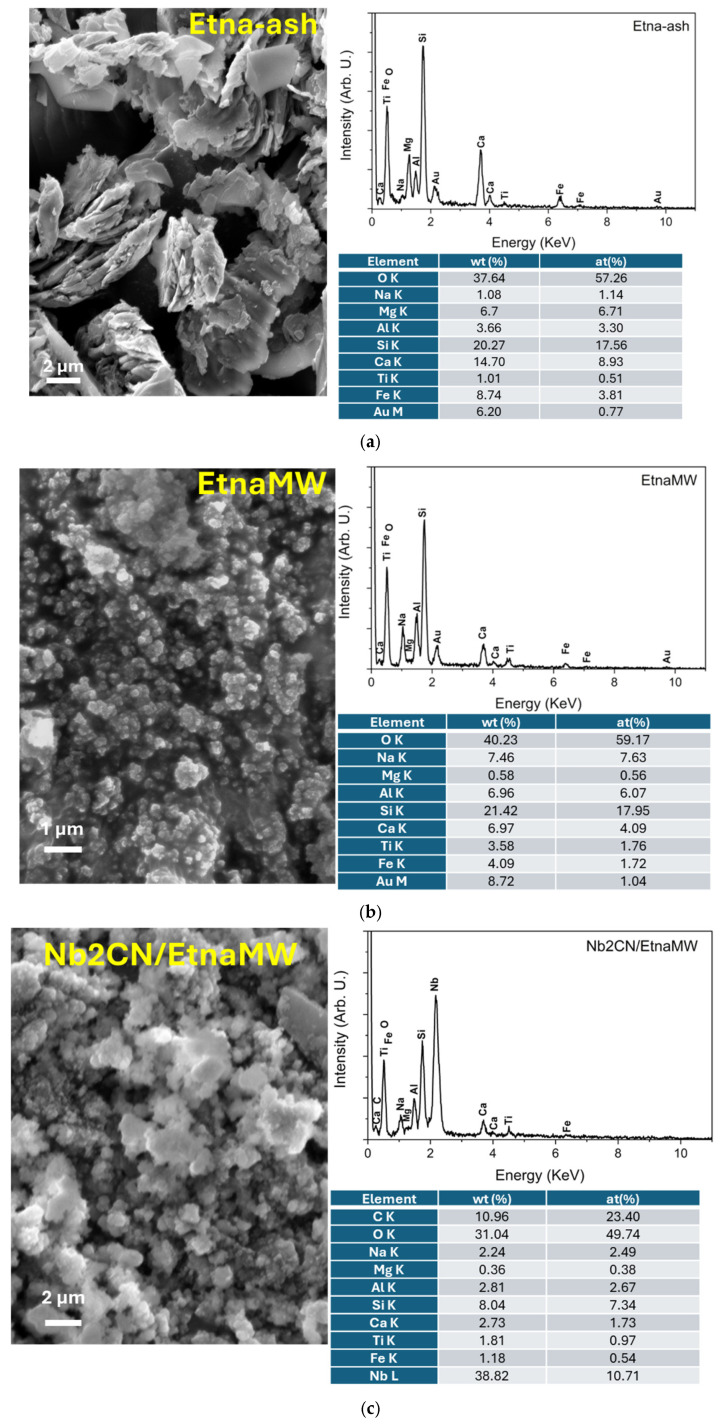

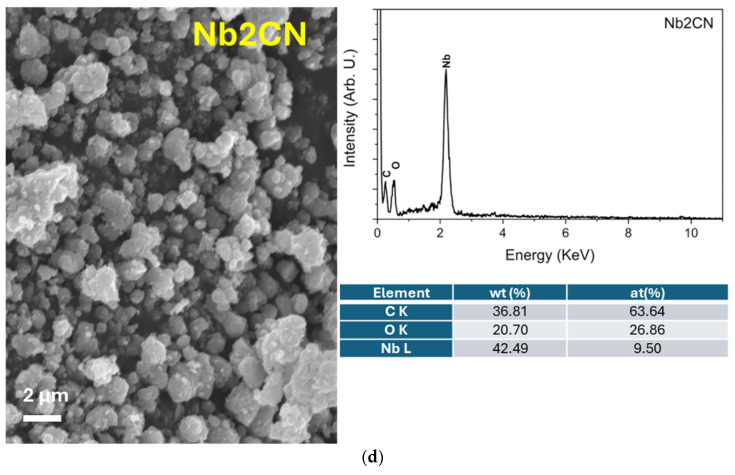
SEM images of the examined samples: (**a**) Etna-ash; (**b**) EtnaMW; (**c**) Nb2CN/EtnaMW; (**d**) Nb2CN. The presence of Au in some samples was related to the coating pre-treatment (see Section 2.2), whereas in the CN-based samples the signal of nitrogen (of carbon nitride) overlapped with the C and O K-alpha signals.

The combined acid washing and microwave irradiation treatments induced a marked modification of the material (EtnaMW), as evidenced by both the XRD pattern and the SEM-EDX analyses. In particular, the EtnaMW sample showed a clear attenuation, or disappearance, of several reflections observed in the raw ash, indicating the selective removal of part of the secondary crystalline phases originally present. This behavior is particularly evident in the low and mid 2θ angular regions, where the contributions attributable to Ca-, Mg-, and Fe-rich mineral components are strongly reduced. This interpretation is consistent with the EDX data (Figure 2) showing, after treatment, a drastic decrease in Mg content (from 6.7 to 0.58 wt%), accompanied by a significant decrease in Ca (from 14.70 to 6.97 wt%) and Fe (from 8.74 to 4.09 wt%). At the same time, a relative increase in the contributions of Na, Al and Si was observed, suggesting that the treatment did not promote the formation of new crystalline phases but rather a selective demineralization of the ash with removal of the Mg, Ca and Fe phases. In this context, the diffraction pattern of the treated ash sample can reasonably be mainly associated with Na-Al-Si or Na-Ca-Al-Si feldspathic-type phases.

SEM observations (Figure 2) further support the modification of the material. Indeed, compared with the lamellar and irregular morphology observed for the Etna ash, the EtnaMW sample displays a much finer, more aggregated and more homogeneous texture, indicative of substantial microstructural reworking induced by the MW treatment. The morphological change, together with the macroscopic color change of the sample (Figure S1, from the black of the bare ash to the beige of the EtnaMW-based samples), is consistent with the partial dissolution and removal of some mineral components, likely including Fe-rich phases.

The niobium oxide and the Nb2CN composite showed the typical Nb_2_O_5_ amorphous pattern related to the Nb-based oxides calcined at temperatures lower than 500 °C [21]. The addition of 2 wt% of g-C_3_N_4_ did not significantly alter the XRD patterns of the bare Nb_2_O_5_, due to the very low amount of g-C_3_N_4_ in the composite [21]. The Nb_2_CN/EtnaMW sample therefore exhibited a pattern similar to that of EtnaMW, with small modifications related to the presence of the amorphous nature of Nb2CN.

The morphology and the elemental composition determined by SEM-EDX of the samples are displayed in Figure 2. The Etna ash showed its typical morphology, already reported, characterized by large interconnected vesicles with complex and/or irregular shapes (Figure 2a) [26,27]. The combined chemical and microwave-assisted treatments induced a structural rearrangement, in accordance with XRD, leading to the formation of irregular porous aggregate particles (Figure 2b), which are more clearly observable in the magnified SEM images of the EtnaMW sample (Figure S2). Such a morphology is commonly associated with the presence of dispersed or supported metal oxide phases, including TiO_2_-based and mixed metal oxide composites [28,29,30,31]. The further addition of the Nb2CN composite led to the appearance of interconnected spherical particles usually related to the Nb_2_O_5_ (Figure 2c,d) [21,32], whereas the sheets of g-C_3_N_4_ are not visible, probably due to their the low amount (2 wt%) and to their coverage by the Nb_2_O_5_ particle agglomerates, as also reported in the literature [21,32].

As already discussed, the changes in morphology reflect a change in the chemical composition of the samples. Indeed, after the ash acidic washing step, a decrease in the alkaline/basic elements was verified. Furthermore, microwave-assisted crystallization of EtnaMW induced phase segregation and agglomeration of Ti species, likely in the form of TiO_2_ particles, resulting in an increase in Ti content from 1.0 wt% in the Etna ash to 3.6 wt% in EtnaMW. In contrast, the Si/Al ratio decreased from approximately 5 in the Etna ash to about 3 in the EtnaMW sample (Figure 2a,b). These features are consistent with the formation of mixed oxide composites based on TiO_2_-FeO_x_ supported on Na/Ca-aluminosilicate. The change in morphology promoted the formation of this type of mixed metal oxide composite as well as the introduction of porosity and a structural rearrangement, as detected by XRD and SEM (Figure 1 and Figure 2) [33,34]. The addition of Nb2CN led to the exploitation of the EtnaMW sample as a support with the homogenous and preponderant distribution of Nb2CN on the aluminosilicate structure (Figure 2c), as also revealed by the SEM-EDX maps (Figures S3 and S4).

The FTIR spectra of the same samples are reported in Figure S5. All the samples showed wide bands in the ranges 3200–3500 cm^−1^ and 1610–1630 cm^−1^ assigned to the stretching and bending vibrations of the -OH bonds respectively, related to the presence of physiosorbed water, whereas for the Etna ash-derived composites, these bands were slightly shifted, especially those at high wavenumbers, due to the presence of silanol end-groups (Si–O–H and Si–OH–Al) and aluminum vibrations (AlOH–) of the aluminosilicate structures [35]. In the latter samples, the bands at about 1020 and 860 cm^−1^ related to the stretching and bending vibrations of the Si-O-Si bonds were also visible [36]. It is possible to note for the Etna-ash sample a negative feature at about 1360 cm^−1^ likely associated with the desorption of physiosorbed atmospheric CO_2_ (Figure S5) [37]. Conversely, in the EtnaMW sample the bands at about 1411 cm^−1^ and 1307 cm^−1^ are assigned to the formation of noncoordinated carbonate (ν_as_ CO_3_^2-^) and monodentate carbonate (ν_s_ COO^-^) respectively [38]. These species are reasonably formed due to the adsorption of the atmospheric CO_2_ on the rearranged Na-Al-Si structures of the EtnaMW, as detected by XRD, pointing to the formation of stronger basic sites compared to the Etna-ash with an improved basicity.

In the Nb2CN sample, the broad envelope of the bands in the range 1490–1200 cm^−1^ at a low intensity (due to the low amount) is ascribed to the presence of the graphitic carbon nitride [22,39]. In particular, the stretching vibrations of aromatic heptazine derived from repeating units as C-H, O-H, C-O, and C-O-C bonds are typically associated with the signals between 1200 and 1450 cm^−1^, whereas the C=N amide bond is consistent with the signal at 1490 cm^−1^ [22,39]. These signals were present also in the Nb2CN/EtnaMW, confirming the interaction between the Nb2CN composite and the EtnaMW. Finally, the bands at 1034 cm^−1^ and the wide one centered at about 610 cm^−1^ in the Nb_2_O_5_ and Nb2CN samples were assigned to Nb=O and Nb-O group vibrations, respectively [40]. Notably, the spectra of the EtnaMW and Nb2CN/EtnaMW samples are quite different in the range of 800–600 cm^−1^ compared to the Etna-ash. This is the typical zone of the metal–oxygen bonds, and it is a further confirmation that in the treated samples the metal oxide species and the Nb-based composite effectively interact with the formed aluminosilicate structures.

**Figure 3:**
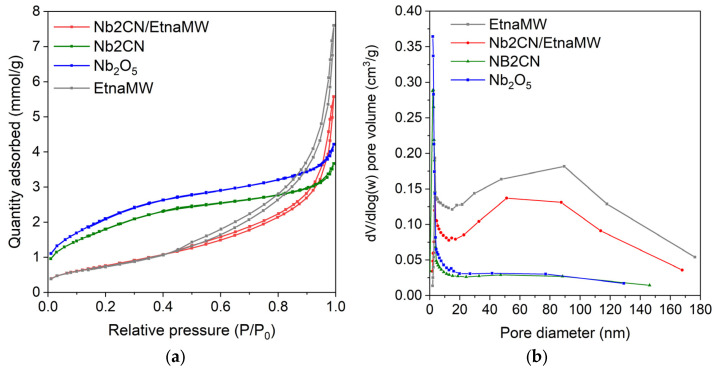
Textural properties of the examined samples: (**a**) N_2_ isotherm curves; (**b**) BJH pore size distribution (desorption curves).

Other important changes due to the chemical and microwave treatments were found in the textural properties, as expected (Figure 3 and Table 1).

As observed from the SEM morphologies (Figure 2), the modification of the Etna ash induced porosity in the material. Indeed, the surface area of EtnaMW was 64 m^2^/g, whereas the raw ash did not exhibit appreciable textural properties (Table 1). The addition of the Nb2CN composite did not change the specific surface area, with a similar pore volume and pore size distribution (Figure 3b and Table 1). These samples (EtnaMW and Nb2CN/EtnaMW) showed a type III isotherm with a H3 hysteresis loop (Figure 3a) usually associated with the presence of slit-shaped pores [41]. On the contrary, the Nb2CN and the Nb_2_O_5_ exhibited a much larger surface area (Table 1) with a type II isotherm with a little H4 hysteresis associated with narrow slit-like pores [41]. These latter samples were also characterized by a narrow pore size distribution and mean pore diameters of about 4 nm (Figure 3b and Table 1), whereas the EtnaMW-based composites showed larger pores (about 10 nm), a higher pore volume (Table 1) and the presence of macropores (pores > 50 nm; Figure 3b).

It is important to note that the adsorption step observed immediately near zero P/P_0_ for Nb_2_O_5_ and Nb_2_CN and to a lesser extent for Nb_2_CN/EtnaMW and EtnaMW indicates the presence of some microporosity in these samples. The possible influence of microporosity on photocatalytic activity, including surface adsorption and mass-transfer effects, will be systematically investigated in future works.

The UV-DRS spectra of the samples are reported in Figure S6, whereas the estimated optical bandgap is reported in Table 1. Interestingly, EtnaMW showed a large feature in the range of 300–500 nm, reasonably associated with the presence of the photo-active metal oxides (TiO_2_-FeO_x_ composites) in the aluminosilicate structure that led to a similar bandgap (3.0–3.2 eV) in the pure semiconductors as TiO_2_ or Fe-doped TiO_2_ [42]. Although these oxides were also present, in different amounts (Figure 2), in the Etna-ash, the untreated ash did not show optical properties. Probably the different morphology (Figure 2) and the structural arrangement of the metal oxides in the bare ash did not allow efficient exploitation of their optical properties, and for this reason the chemical and microwave-assisted treatments were necessary. The addition of Nb2CN slightly modified the optical properties, with, however, a comparable bandgap compared to the EtnaMW (Table 1). The bare Nb2CN and Nb_2_O_5_ exhibited a bandgap of 3.4 eV (Table 1) typical of Nb oxide-based samples [43].

### 3.2. Solar Photocatalytic H_2_ Production

The samples were tested in the solar TEOA (a common holes scavenger) photoreforming. A preliminary photocatalytic screening evaluated the influence of g-C_3_N_4_ addition on the as-prepared niobium oxide. As shown in Figure S7, a loading of 2 wt% of carbon nitride provided the optimal enhancement in performance compared to the bare Nb oxide. In contrast, as widely reported in the literature, bare g-C_3_N_4_ did not exhibit detectable H_2_ production due to the rapid recombination of its photogenerated charge carriers [21,22].

An efficient heterojunction between Nb_2_O_5_ and g-C_3_N_4_ is reasonably achieved only at an optimal, low carbon nitride loading (2 wt%). Higher loadings likely promote charge carrier recombination covering the active sites of niobium oxide, while lower amounts (< 2 wt%) are insufficient to ensure effective interfacial interaction and optimal separation of photogenerated electron–hole pairs. On this basis, the Nb2CN composite was subsequently combined with the EtnaMW material.

**Figure 4:**
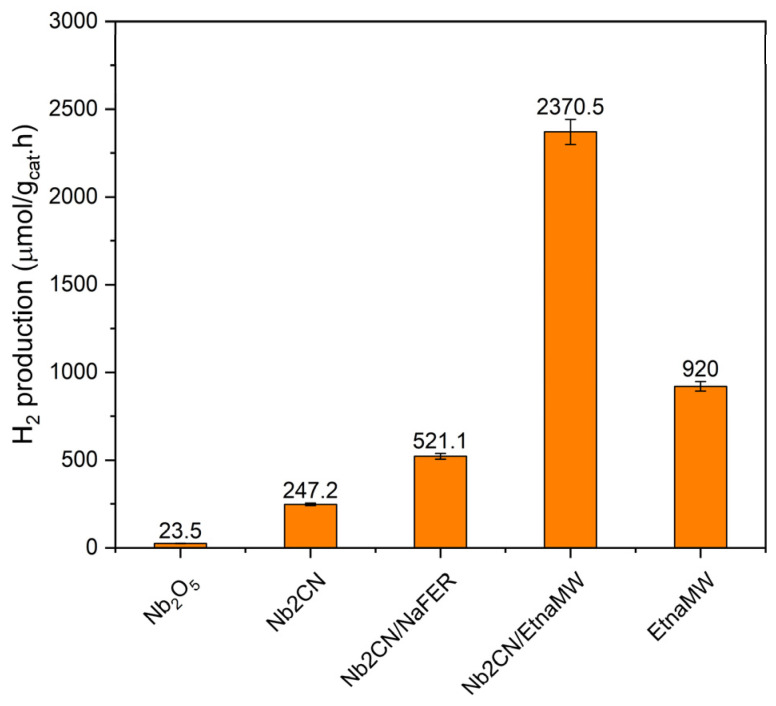
H_2_ production rate for the examined samples in the solar TEOA photoreforming.

As shown in Figure 4, the synergistic interaction between Nb2CN and EtnaMW results in a H_2_ production rate significantly higher than the sum of the individual contributions of the two components. To further verify the specific role of the EtnaMW matrix, the same Nb2CN composite was also combined, following the procedure described in Section 2.1.3, with a commercial zeolite support (Zeolyst™ ferrierite, FER, Na-form; specific surface area ≈ 200 m^2^/g). Notably, the use of a photocatalytically inert support such as this zeolite, despite its high surface area, resulted in inferior perfomance compared with the bare EtnaMW (Figure 4). This result indicates that, under the adopted experimental conditions, the photocatalytic performance is not governed exclusively by the surface area. Indeed, bare Nb_2_O_5_ and Nb2CN, which have higher specific surface areas (177 and 150 m^2^/g, respectively), compared with the EtnaMW-based samples (64–66 m^2^/g, Table 1), showed significantly lower H_2_ evolution rates (Figure 4). Nevertheless, the contribution of some microporosity, present in all the samples, expect for the bare ash, cannot be excluded, particularly in relation to adsorption phenomena and mass-transfer effects during photoreforming.

Importantly, the untreated Etna ash did not exhibit photocatalytic activity, as expected, because of the lack of suitable optical and textural properties. The combined chemical and microwave-assisted treatments are therefore essential to induce structural rearrangement of the aluminosilicate framework and generate meso- and macroporosity (Figure 3, Table 1), improving reagent accessibility and surface interactions during solar photoreforming. At the same time, these treatments promote changes in the distribution and relative abundance of the naturally occurring Ti- and Fe-based species within the aluminosilicate matrix. In particular, the Ti/Fe ratio increased from approximately 0.1 in raw Etna ash to about 0.9 in EtnaMW (Figure 2). This compositional rearrangement favors the formation of mixed TiO_2_–FeO_x_ photoactive domains in optimal amounts within the aluminosilicate structure. Indeed, according to the literature, photocatalytic H_2_ evolution is enhanced when TiO_2_ is the primary component of a composite or when it is finely dispersed on a support (at loadings ≤ 10 wt%), enabling improved exposure of active sites and more efficient charge transfer processes [23,44]. Conversely, iron oxides generally exhibit lower intrinsic photocatalytic activity and more commonly act as dopants or co-catalysts that facilitate interfacial charge separation [45,46].

Consequently, the good photocatalytic performance of the EtnaMW sample can be related to the elemental composition variation of these metals (Ti and Fe) on the aluminosilicate structure (about 7–9 wt%). The subsequent incorporation of Nb2CN allowed EtnaMW to act as a photoactive support, leading to the development of a multifunctional and high-performance photocatalyst. After the absorption of the UV-A portion of the incident solar light and the consequence formation of the electrons–hole pairs, the strong interaction between the metal oxides and the aluminosilicate structure of the EtnaMW and the further presence of the Nb2CN resulted in an extended lifetime of the e^−^/h^+^ pairs, with the possible occurrence of band bending at the interfaces compared to the bare Nb_2_O_5_ and Nb2CN [47]. As a result of these synergistic effects, the H_2_ production rate of Nb2CN/EtnaMW was approximately 2.5 and 9.5 times higher than those of EtnaMW and Nb2CN alone, respectively.

The photocatalytic stability of the Nb_2_CN/EtnaMW sample was evaluated by performing six consecutive runs of solar TEAO photoreforming (Figure S8). At the end of each 5 h run of simulated solar irradiation, the catalyst was filtered, dried under vacuum at 80 °C, and reused. As shown in Figure S8, the sample maintained good photocatalytic stability, exhibiting only an approximately 8% loss of activity after the sixth run.

Although a direct comparison of H_2_ production rates reported in the literature is challenging due to variations in the experimental setups, reaction conditions, and irradiation sources among different research groups, Table 2 presents representative data for Nb–CN-based photocatalysts evaluated in solar/visible-light TEOA photoreforming. The peculiar Nb2CN/EtnaMW composite showed an improved H_2_ production rate, similar or higher than those of other Nb/CN-based photocatalysts reported in the literature. The obtained solar H_2_ production rate (2370.5 µmol/g_cat_∙h) is also comparable with other recent solar photocatalytic systems employing g-C_3_N_4_-based photocatalysts, such as gCN doped with B (H_2_ production rate of 1322 µmol/g_cat_∙h using 0.1 M Na_2_S and 0.1 M Na_2_SO_3_ solution as the hole scavenger) [48], heterojunctions formed with gCN/Pt/TiO_2_ (H_2_ production rate of 560 µmol/g_cat_∙h using glucose as the sacrificial agent) [49], and other Nb-based composites such as Nb_2_CT_x_ MXene/TiO_2_ (800 µmolH_2_/g_cat_∙h with ethanol/water solution) [50] and Na_0.5_Bi_2.5_Nb_2_O_9_ perovskite (242 µmolH_2_/g_cat_∙h with H_2_PtCl_6_·6H_2_O (0.25 M)/water solution) [51].

On the solar photocatalytic performance of the Nb2CN/EtnaMW catalyst, the apparent quantum efficiency (AQE%) was also estimated considering the 315–400 nm spectral range, the employed experimental setup (see Section 2.3), and the calculation procedure reported in ref. [21] and in the supporting information. The Nb2CN/EtnaMW catalyst exhibited an AQE of approximately 4.2%. For comparison, the Nb_2_O_5_-gC_3_N_4_ photocatalyst reported in the same reference showed an AQE of about 14% under UV-LED irradiation [21]. The lower AQE observed for Nb2CN/EtnaMW is reasonably attributable to the different irradiation source and experimental configuration adopted in the present work, namely, the use of a solar simulator with optical fiber instead of UV irradiation. Nevertheless, Nb2CN/EtnaMW exhibited a significantly higher H_2_ production during the solar TEOA photoreforming, with a H_2_ evolution rate approximately three times higher than that reported for Nb_2_O_5_-gC_3_N_4_ (Table 2, entries 1 and 2). This points to the need for more efforts to improve the optical features of the Nb2CN/EtnaMW. Further investigations will be devoted to clarifying the reaction mechanism and the band configuration at the interfaces of the complex EtnaMW-based material, such as electrochemical characterization measurements, photoluminescence (PL) spectroscopy, transient photocurrent response, electrochemical impedance spectroscopy (EIS), etc. These studies will provide deeper insight into the electronic interactions occurring within the Etna-MW matrix and may contribute to further improvements in AQE.

Finally, adopting the same photocatalytic experimental conditions (see Section 2.3), we tested the performance of the bare TiO_2_ P25 (P25 Aeroxide surface area 50 m^2^/g, <100 nm of particle size, as purchased from Acros Organics) that showed a H_2_ production rate of 25 µmol/g_cat_∙h, similar, in accordance with the literature, to that of the bare Nb_2_O_5_ (Figure 4) [43]. Therefore, compared to TiO_2_ P25, the approximately 95-fold higher H_2_ production rate obtained with Nb_2_CN/EtnaMW is highly promising for the development of a new generation of photocatalysts derived from natural, abundant resources and/or waste materials. The photocatalytically active sites present in EtnaMW, namely, the optimal amount of TiO_2_–FeO_x_ species that strongly interacted with the rearranged aluminosilicate-derived structure, enabled an efficient exploitation of solar activation in the UV-A region. Such behavior is consistent with that reported for iron titanate-based photocatalysts, where the strong electronic interaction between Fe and Ti species promotes enhanced photocatalytic performance and improved charge carrier dynamics [58,59]. The further addition of the Nb2CN composite further enhanced the photocatalytic performance by promoting additional interfacial interactions between the EtnaMW support and the Nb oxide/graphitic carbon nitride heterostructure. This effect improved the charge separation and migration efficiency, ultimately leading to higher hydrogen production rates under simulated solar irradiation. EtnaMW can therefore be effectively combined with conventional and unconventional photocatalysts to further enhance performance while reducing reliance on critical raw materials such as Nb and Ti.

This represents a key advantage for the development of efficient and scalable solutions, promoting sustainability throughout the entire process, from catalyst synthesis to green H_2_ production by exploiting renewable energy sources, such as solar irradiation, together with waste- or naturally derived materials. Nevertheless, the chemical composition of Etna volcanic ash may vary depending on the specific eruption event, sampling location, and geological conditions. Therefore, further optimization of the synthesis protocol is required to minimize the impact of such compositional variability on the physicochemical and photocatalytic properties of the final materials. In this context, microwave-assisted treatments could offer an effective approach to improve material homogeneity and reproducibility, enhancing the scalability and reliability of the proposed catalytic systems.

## 4. Conclusions

Etna ash-derived materials were successfully synthesized through chemical treatment coupled with microwave-assisted crystallization, which promoted the rearrangement of the aluminosilicate framework and introduced porosity into the resulting structure. The obtained materials exhibited remarkable photocatalytic activity for solar-driven H_2_ production due to the presence of optimally distributed photocatalytically active phases, such as TiO_2_ and iron oxides, strongly interacting within the aluminosilicate matrix. The modified Etna ash achieved a H_2_ production rate of 920 µmol/g_cat_∙h and also acted as an effective support for Nb_2_O_5_/g-C_3_N_4_ composites prepared by a hydrothermal method. The coupling between the Etna-derived material and the Nb-based composite generated a pronounced synergistic effect, leading to a hydrogen evolution rate of 2370.5 µmol/g_cat_∙h, significantly higher than those of the individual components. Moreover, the photocatalyst maintained stable activity over six consecutive cycles. These findings demonstrate the possibility of valorizing Etna volcanic ash as a raw material for a new class of multifunctional photocatalysts through an energy-saving microwave-assisted route, avoiding conventional high-temperature treatments. Overall, this work proposes a sustainable circular economy strategy for the conversion of volcanic waste into high-value photocatalytic materials for solar hydrogen production while minimizing the use of noble and critical raw materials.

## Figures and Tables

**Table 1:** Textural and optical properties of the examined samples.

Sample	Specific Surface Area (m^2^/g)	Mean Pore Diameter (nm)	Pore Volume (cm^3^/g)	Eg (eV)
Etna-ash	<1.0	/	/	/
EtnaMW	64.0	10.1	0.26	3.0
Nb2CN/EtnaMW	66.0	9.4	0.20	3.2
Nb2CN	149.3	3.9	0.12	3.4
Nb_2_O_5_	177.2	3.9	0.14	3.4

**Table 2:** Solar/visible photocatalytic H_2_ production comparison data on solar TEOA photoreforming with Nb-CN-based photocatalysts.

Sample	Experimental Conditions	Irradiation Source	H_2_ Production (µmol/g_cat_∙h)	Ref.
Nb_2_CN/EtnaMW	50 mL of TEOA aqueous solution (20 *v*/*v*%)	Xe lamp 150 W, 10.0 mW/cm^2^∙nm optical fiber solar simulator	2370.5	This work
Nb_2_O_5_-gC_3_N_4_	4% (*w*/*w*) TEOA aqueous solution	Natural solar light	810	[21]
Nb_2_O_5_/La_2_O_3_	100 mL aqueous solution consisting of 90 mL of water and 10 mL of TEOA	300 W Xe lamp	2175	[52]
Co phthalocyanine/CN	10 mL aqueous solution, 50 μL of 8 wt% H_2_PtCl_6_ solution and 2 mL of TEOA	Xe lamp 300 W, 100 mW·cm^−2^ with UV cut-off filter (λ ≥ 420 nm)	1136.5	[53]
Cu complex carbon nanotubes/CN	100 mL TEOA solution (10 *v*/*v*%)	Xe arc lamp 300 W, 100 mW·cm^−2^ with UV cut-off filter (λ ≥ 420 nm)	931	[54]
Ni(OH)_2_/NiO/Ni-CN	TEOA solution (30 *v*/*v*%)	430 nm LED 50 mW/cm^2^	1200	[55]
CN from urea/CN from melamine/Pt-TiO_2_	90 mL deionized water, 10 mL TEOA	Xe lamp 300 W, 257.2 mW/cm^2^ coupled with a 420 nm cut-off filter	1735	[56]
Pt/C-doped CN	100 mL TEOA solution (10 *v*/*v*%)	300 W xenon lamp with a long-pass filter of (λ ≥ 420 nm)	2192.2	[57]

## Data Availability

The original contributions presented in this study are included in the article/Supplementary Materials. Further inquiries can be directed to the corresponding authors.

## Supplementary Materials

The following supporting information can be downloaded at https://www.mdpi.com/article/10.3390/ma19112240/s1: Table S1: Etna ash composition; Figure S1: Sample photos; Figure S2: SEM images magnification of the EtnaMW sample; Figure S3: SEM-EDX map of the EtnaMW; Figure S4: SEM-EDX map of the Nb_2_CN/EtnaMW; Figure S5: FTIR spectra of the examined samples; Figure S6: UV-DRS spectra of the examined samples; Figure S7: H_2_ production rate of the NbCN samples varying the amount of carbon nitride; Figure S8: H_2_ production rates obtained during different runs of solar TEOA photoreforming employing the Nb_2_CN/EtnaMW sample; Text S1: Estimation of the apparent quantum yield (AQE%).
