# Supplementary material for: Nb2O5/g-C3N4 Composite Photocatalysts Supported on Etna-Derived Aluminosilicate for Solar H2 Production
Source: Materials (Basel). 2026 May 26;19(11):2240. doi: 10.3390/ma19112240 (PMC13257617; doi:10.3390/ma19112240)
Supplement: Supplementary file 1 [file materials-19-02240-s001.zip › materials-4314444-supplementary.pdf]

Supporting information

- **Etna ash composition**

Table S1. Furnished chemical composition (determined by WD-XRF) of the Etna ash sample used in this study.

Species	Chemical Composition (wt%)
SiO_2	46.87
Al_2O_3	16.16
FeO_{tot}	11.49
CaO	10.82
MgO	5.59
Na_2O	3.32
TiO_2	1.91
K_2O	1.89
L.O.I.[1]	1.28
P_2O_5	0.46
MnO	0.21

[1] Loss on ignition determined by gravimetric methods.

- **Sample photos**

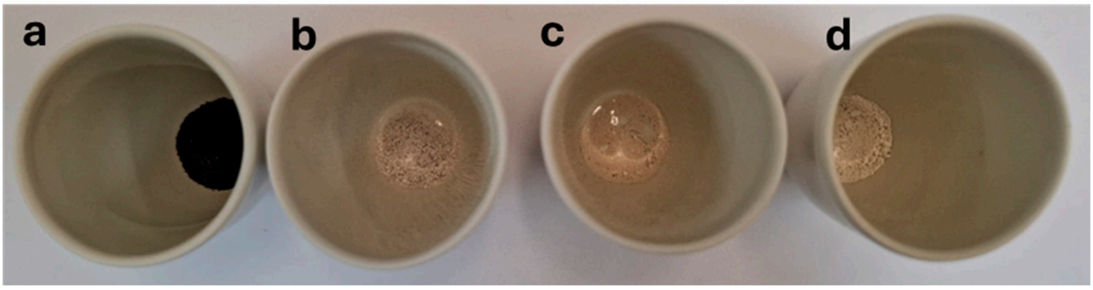

Figure S1. Photos of the examined powders: (**a**) Etna ash; (**b**) EtnaMW; (**c**) Nb_2CN/EtnaMW; (**d**) Nb2CN.

- **SEM image magnification of the EtnaMW sample**

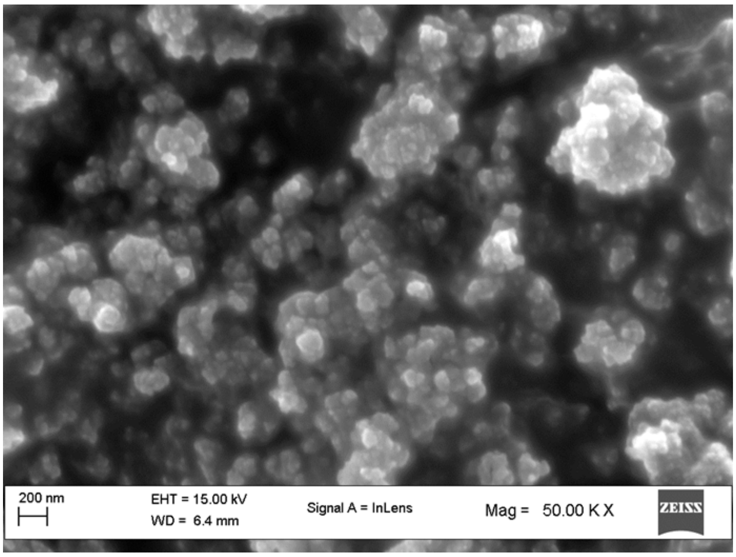

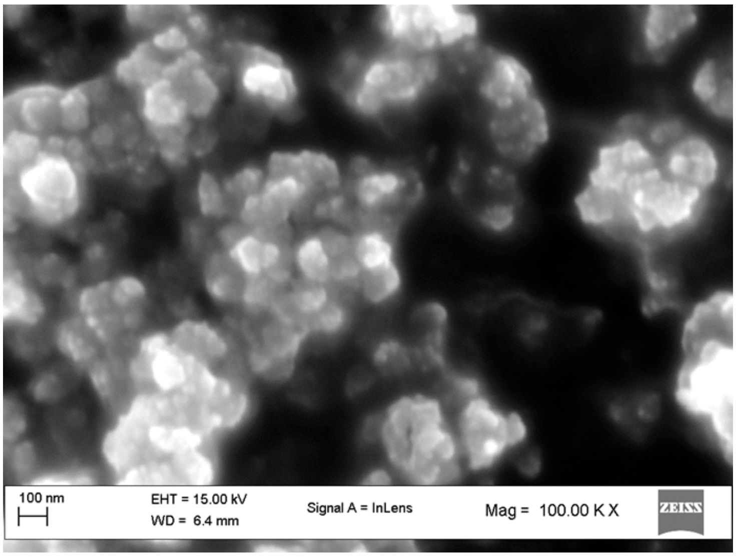

Figure S2. SEM images of the EtnaMW sample.

- **SEM-EDX map of the EtnaMW**

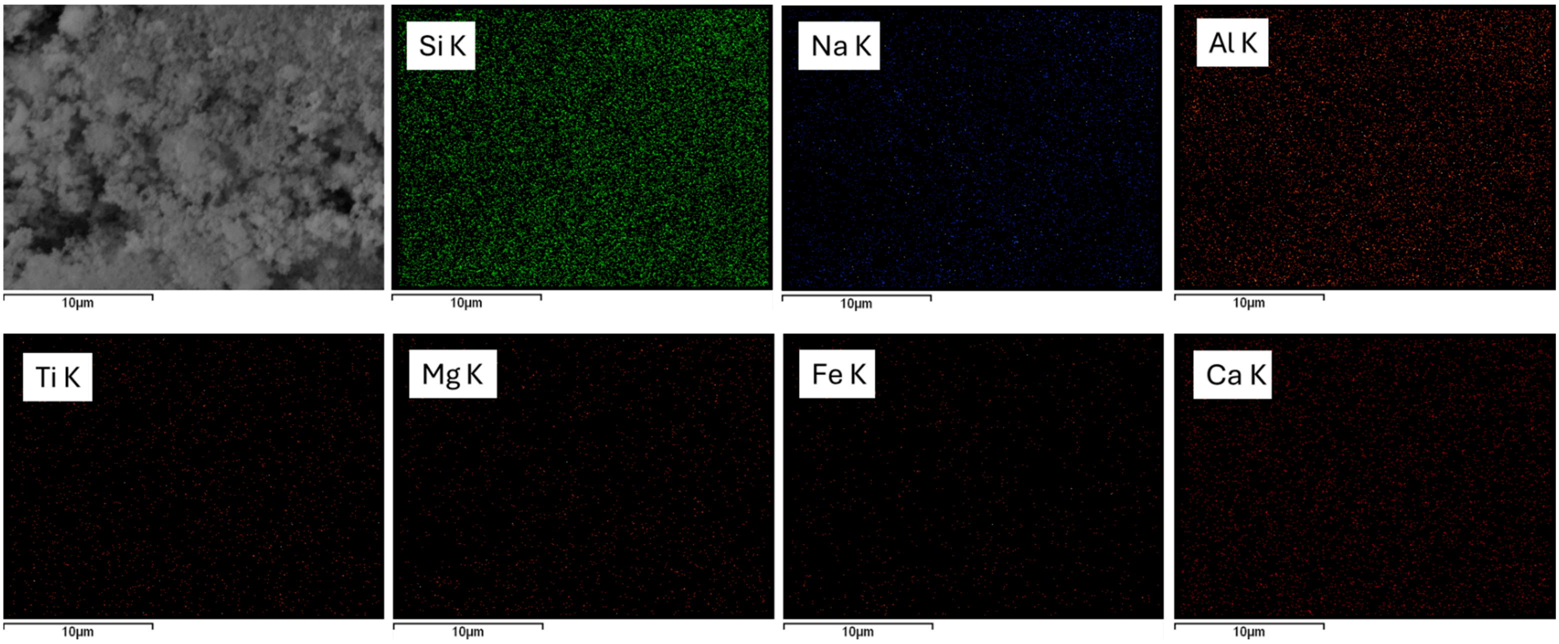

Figure S3. SEM-EDX map of the EtnaMW sample.

- **SEM-EDX map of the NB2CN/EtnaMW**

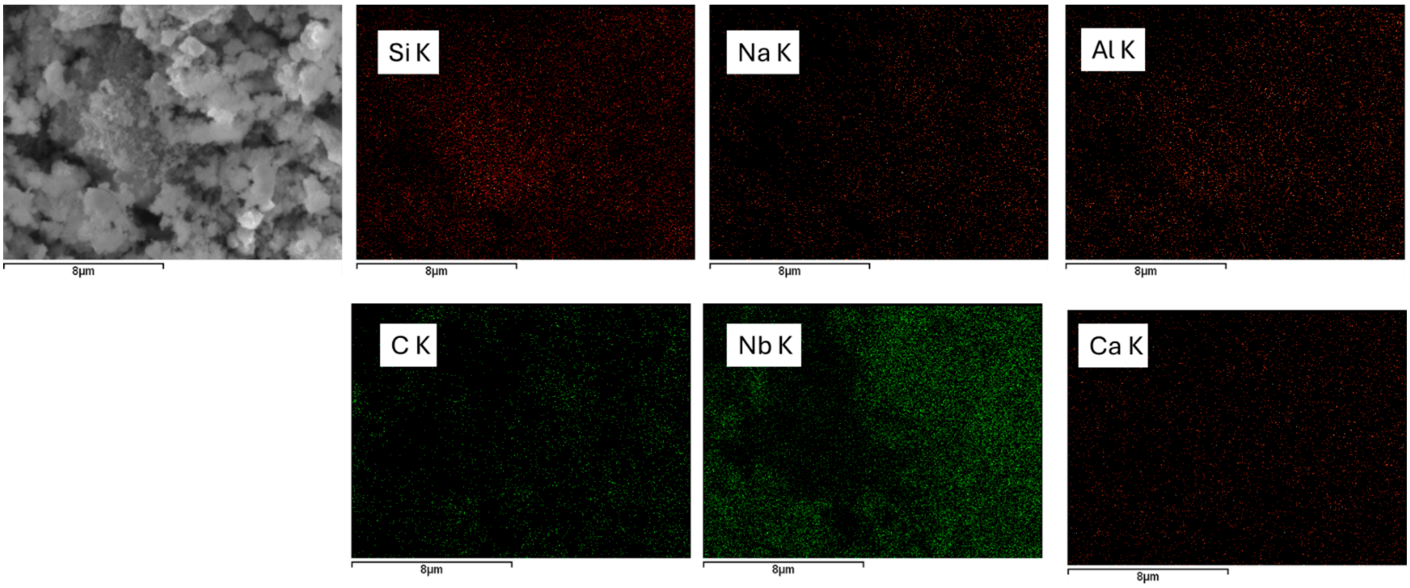

Figure S4. SEM-EDX map of the Nb_2CN/EtnaMW sample.

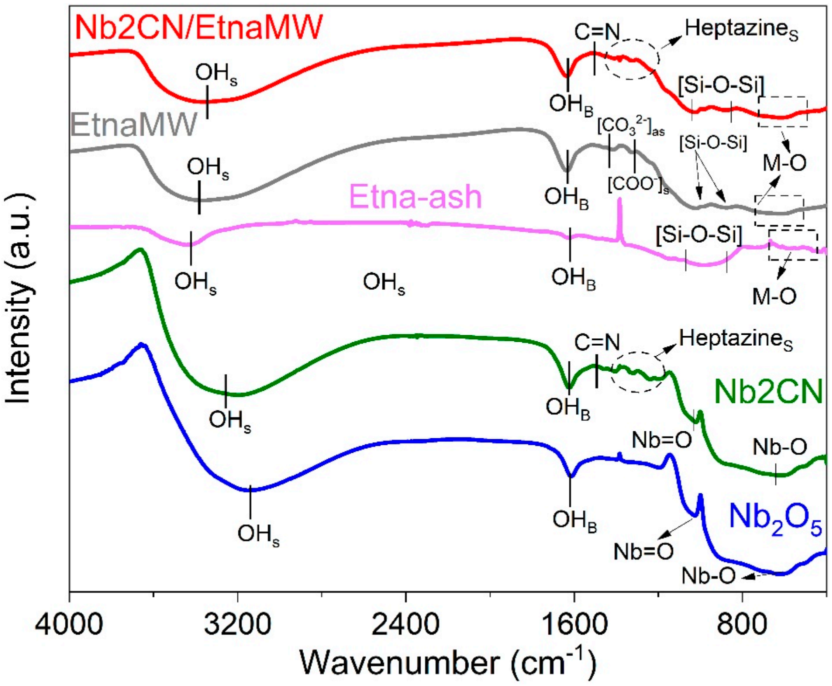

Figure S5. FTIR spectra of the examined samples.

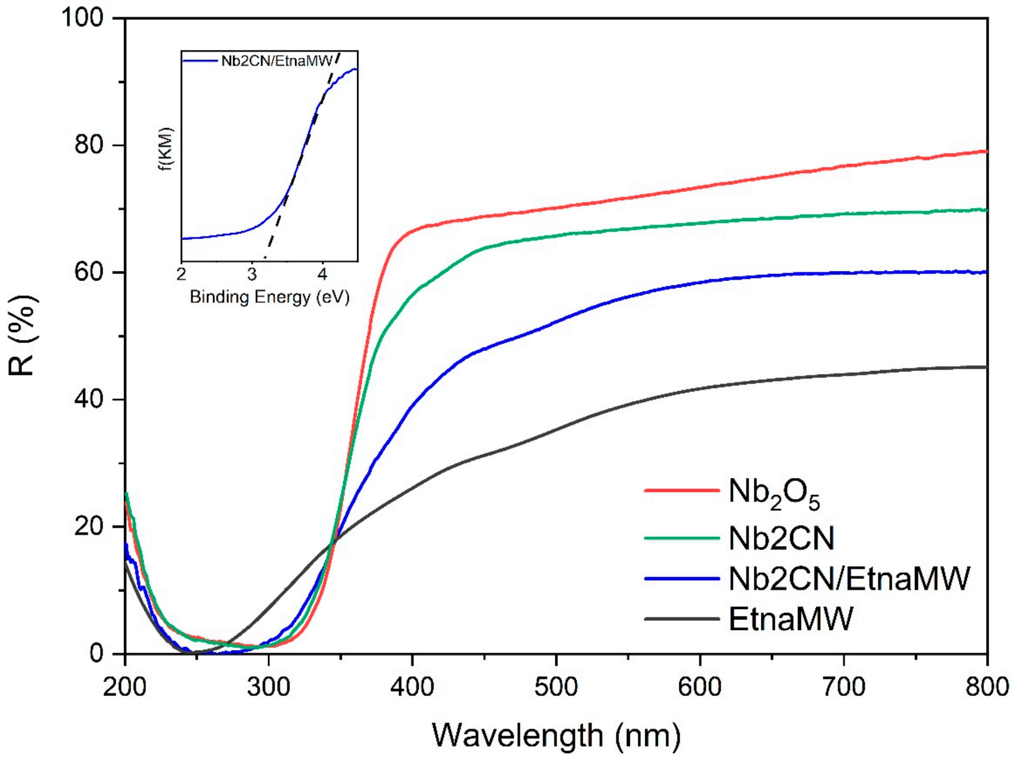

Figure S6. UV-DRS spectra of the examined samples. Inset is the estimation of the optical bandgap by plotting the modified Kubelka–Munk function vs the binding energy for the Nb_2CN/EtnaMW as a representative sample.

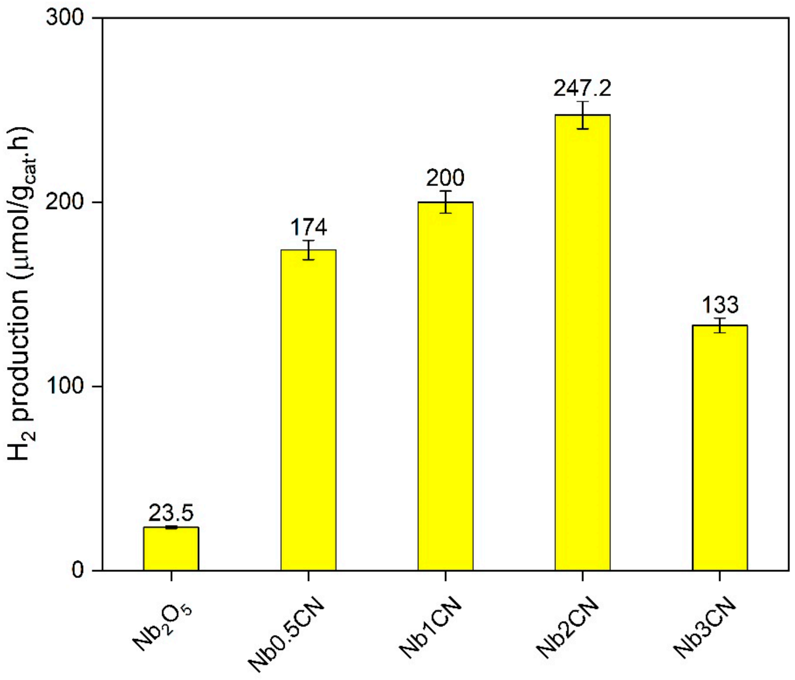

Figure S7. H_2 production rate of the examined samples in the solar TEOA photoreforming.

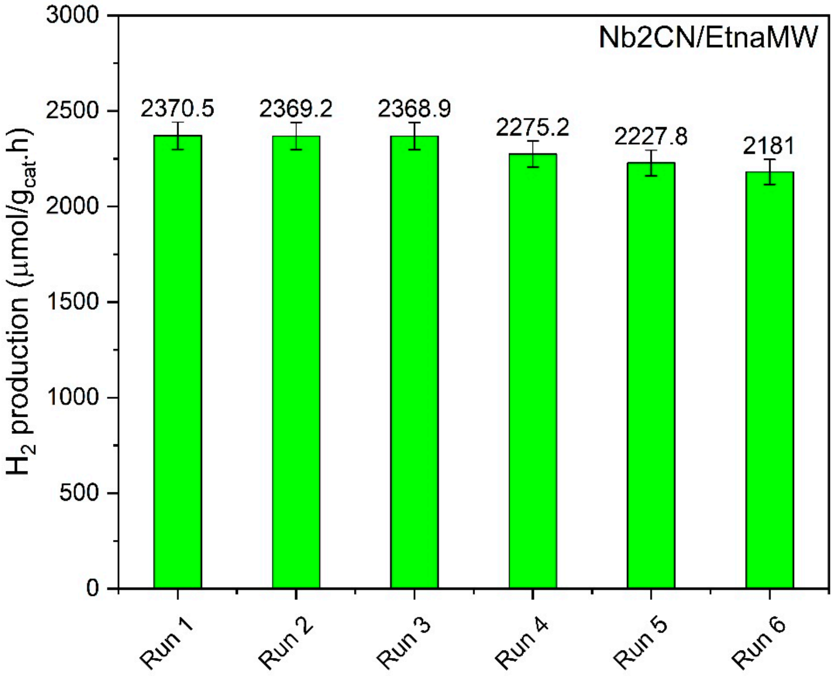

Figure S8. H_2 production rates obtained during different runs of solar TEOA photoreforming employing the Nb2CN/EtnaMW sample. Each run was 5 h of simulated solar irradiation. At the end of each run the catalyst was filtered, dried under vacuum at 80 °C and reused.

Text S1

- **Estimation of the Apparent Quantum Yield (AQE%)**

The estimation of the AQE was made considering the H_2 production rate in the solar TEOA photoreforming (2370.5 µmol/ g_{cat}·h), the experimental set-up reported in Section 2.3 (irradiance of 10.0 mW/cm^2·nm; UV-A spectral range: 315-400 nm, consistent with the E_g of the sample; see Table 1), with 40 mg of the employed catalyst and a 0.5 cm^2 irradiated area.
The applied formula is as follows [1]:

$$AQE(\%) = \frac{2 \times moles\ of\ evolved\ H_2}{moles\ of\ incident\ photon} \times 100$$

References:

1. García-López, E.I.; Genco, A.; Lagostina, V.; Paganini, M.C.; Marcì, G. Photocatalytic generation of H2 by photoreforming of organics in aqueous suspension of Nb2O5/C3N4 composites. *Catal. Today* **2023**, *423*, 114283, doi:10.1016/j.cattod.2023.114283.
